# Epidemiology of *Leptospira* sp. Infection: Current Status, Insights and Future Prospects

**DOI:** 10.3390/microorganisms12010022

**Published:** 2023-12-22

**Authors:** Sérgio Santos de Azevedo

**Affiliations:** Academic Unit of Veterinary Medicine, Center for Rural Health and Technology, Federal University of Campina Grande, Patos 58708-110, Brazil; sergio.santos@professor.ufcg.edu.br

In recent decades, the scientific community has been faced with an increased risk of emerging or re-emerging zoonotic diseases, such as leptospirosis, mainly originating from anthropic actions [1]. In this scenario, the One Health initiative has become a strong and global multidisciplinary approach to understanding and controlling diseases with well-known human–animal–ecosystem interactions [2].

Leptospirosis is deemed a reemerging neglected infectious disease having important economic impacts on livestock production and representing a significant public health issue [3]. Despite promising research focusing on vaccine improvement in recent decades, commercially available vaccines do not confer suitable immunity [4]. The causative agent is pathogenic *Leptospira* spp., and the broad number of serovars, animal hosts and transmission routes (directly via contact with infected animals or indirectly via water and soil contaminated with the agent) make the infection one of the most highly distributed worldwide [5,6].

The reservoirs of infection are rodents and small marsupials, and dogs, cattle and pigs are also important sources of infection [7]. Mainly in tropical countries, people are in close contact with environments inhabited by *Leptospira* spp. Carriers, and therefore, they are more exposed to the risk of infection [8]. Due to the complex epidemiology and ecology, wide range of susceptible hosts and reservoirs, and strong environmental component, the infection must be studied from the One Health point of view in order to bridge gaps and contribute to a broader understanding of the dynamics of infection, with the goal being improvements in infection control and prevention.

In recent years, efforts have been made to promote innovations mainly regarding the epidemiology, diagnosis, control and pathogenesis of leptospirosis in the One Health context. Santos et al. [9] reported the first structure of the methyl-accepting chemotaxis protein CACHE domain from *L. interrogans*, which demonstrated similarity to other bacterial chemoreceptors of motility and chemotaxis. Gold nanoparticles (AuNPs) have been shown to be promising in medicine, and the results from the paper by Sooklert et al. [10] reported the possible immunotherapeutic use of AuNPs in the *Leptospira*-induced TLR2-mediated innate immunity. A live attenuated mutant vaccine based on a motility-deficient mutant lacking the expression of a flagellar protein, FcpA, has been suggested to promote protection against distinct pathogenic *Leptospira* spp. [11]. It is very well known that the major challenge in the control of leptospirosis is to develop vaccines able to confer protection against all pathogenic serovars, prevent renal colonization, and induce long-lasting immune protection. In this way, rLIC13259 and rLIC11711 proteins have been highlighted as having greater ability to control leptospire colonization in hamster kidneys [12]. 

Pathogenic *Leptospira* spp. have also been detected in environmental samples. Miller et al. [13] found pathogenic leptospires in 4 (3.7%) of 112 river samples, as well as in 22% of shore soil and 16.7% of soil samples in the transects. In addition, the storage of contaminated water samples for 2 to 4 weeks in the dark at an ambient temperature prior to microbiological culture improved pathogenic *Leptospira* spp. isolation [14].

Bovine genital leptospirosis (BGL) has been proposed as a dissociated syndrome from renal/systemic disease [15]. Evidence from studies conducted in semiarid areas in livestock (bovine, sheep, swine and goats) have shown that even in the adverse environmental conditions of a semiarid region, leptospires may survive and propagate by the genital alternative route of transmission [16,17,18,19,20]. These findings are important because in such areas the indirect transmission routes are impaired due to the adverse climate conditions for *Leptospira* spp. survival in the environment.

This Special Issue includes a collection of studies focusing on the analysis of phenotypic and genotypic characteristics of *Leptospira* spp. strains isolated from infected people in Slovenia using both traditional and modern methods; the detection of *Leptospira* spp. and anti-leptospiral antibodies in donkeys from a zoonosis center in the semiarid region of Brazil through the paired collection of biological material; the development and evaluation of a molecular diagnostic tool focused on the improvement in diagnosis of *Leptospira* spp. infection in different types of biological samples, including those ones collected after antimicrobial therapy against leptospirosis; and a cross-sectional survey for *Leptospira* spp. using serological and molecular methods in stray dogs and cats from Italy. In addition, there are three review papers focusing on the relevance of *Leptospira* spp. infection, highlighting aspects regarding its diagnostic negligence when compared with dengue and malaria in Colombia, the need for cooperation between several authorities on the study of leptospirosis using the One Health approach in Nordic countries, and some aspects relating to the molecular mechanisms involved in the pathophysiology of leptospirosis, mainly focusing on the role of Na/K-ATPase.

The articles published in this Special Issue present valuable insights into the epidemiology, diagnosis, clinical characteristics, surveillance and pathogenesis of *Leptospira* spp. infection.
